# Comparative effectiveness of PROMPT®-based language training vs. structured home-based training for language and speech delay in children with autism spectrum disorder

**DOI:** 10.3389/fped.2026.1726236

**Published:** 2026-03-26

**Authors:** Wei Hu, Qin Liu, Xuemei Fu, Yanmei Chen

**Affiliations:** Department of Children’s Rehabilitation, Yongkang Women and Children’s Health Hospital, Yongkang City, Zhejiang Province, China

**Keywords:** autism spectrum disorder, children, language and speech delay, language training, PROMPT®

## Abstract

**Objective:**

To compare the relative effectiveness of PROMPT®-based training vs. structured home-based training in facilitating language and speech development in children with autism spectrum disorder (ASD), specifically focusing on how addressing speech motor control impacts overall language stages.

**Methods:**

This retrospective analysis study was conducted at Yongkang Women and Children's Health Hospital. The data for the research subjects were sourced from ASD children with language and speech delay who received treatment from January 2023 to January 2025. To minimize potential confounding and selection bias, a 1:1 propensity score matching (PSM) was implemented using a nearest neighbor algorithm with a caliper of 0.05. Consequently, 62 patients who received PROMPT®-based language training were matched with a cohort of 62 patients receiving structured parent-mediated home training guidance. The Sign-Significant Relation (S-S) and image expression ability were compared before and after the intervention between the two groups.

**Results:**

After the 3-month intervention, the distribution of S-S developmental stages for language comprehension and expression differed significantly between the two groups, with the PROMPT® group showing a higher proportion of children in more advanced stages than the control group (*P* < 0.05). In addition, image expression ability improved in both groups after intervention, and the improvement was greater in the PROMPT® group than in the control group (*P* < 0.05).

**Conclusions:**

PROMPT®-based language training was associated with better outcomes than structured home-based training in children with ASD and language and speech delay, with greater improvement in language developmental stages and image expression ability.

## Introduction

Autism Spectrum Disorder (ASD) is a neurodevelopmental condition that emerges during childhood (1, 2). Its core symptoms comprise social communication deficits alongside restricted and repetitive patterns of behavior or interests (1). Research has found that the global prevalence of ASD is about 0.6% (95% CI: 0.4%–1%), but the prevalence of ASD in China is higher than this level, at 1.8% (95% CI: 1.7%−2.0%), and most ASD children have varying degrees of language and speech delay (3, 4). Language disorders are a form of social communication deficits caused by language and speech delay, which is one of the causes of ASD (5). It is essential to distinguish between language disorders, which involve the cognitive processing of linguistic symbols and rules, and speech disorders, which relate to the motor execution of sounds and articulation (6). In children with ASD, these conditions frequently coexist; they present with delayed language development rooted in social-communication deficits, alongside concomitant speech motor impairments such as poor oral-motor coordination and planning (7). At present, the pathological mechanism of language and speech delay in children with ASD is still unclear. Lee et al. (8) used fMRI and found that there were changes in the language network brain connections in the brain regions of ASD patients, which can lead to difficulties in language and communication. An imaging study has shown that children with ASD have less gray matter and lower activity in their brains, and the connectivity of the prefrontal temporal language network is poor, leading to impaired neural basis for language processing and output (9). Secondly, most children with ASD have problems with oral sensation, exercise planning, and poor coordination of oral muscle groups, which also lead to abnormal vocalization and pitch in ASD children (10, 11). Multiple functional abnormalities combined together cause delayed speech development in ASD patients (8–10). At present, there are no specific targeted drugs for patients with ASD, especially those related to language and speech delay. At present, the main intervention measures include behavioral intervention, speech and language therapy, and social communication training, but most methods pay less attention to the language production process (oral muscle control, speech motor coordination). The Prompts for Restructuring Oral Muscular Phonetic Targets (PROMPT®) technique was originally developed by Deborah Hayden in the 1980s (12). Beyond its tactile-kinesthetic prompts, PROMPT® is grounded in a holistic “Conceptual Mapping” framework that integrates Physical-Sensory, Cognitive-Linguistic, and Social-Emotional domains (13). It uses hand contact with the child's face, lips, and jaw to provide physical guidance and feedback, allowing the child to understand their own oral muscle movement patterns and make corresponding adjustments, promoting coordinated oral muscle movement during pronunciation (14, 15). Recent high-quality evidence, including randomized controlled trials, has validated its applicability and effectiveness in managing severe speech motor delays (15). Furthermore, neuroimaging research has shown that PROMPT® can induce structural neuroplasticity within language-related brain regions, such as Wernicke's area, providing a biological foundation for its impact on linguistic processing (16). Consequently, its systematic application for addressing the specific speech motor planning challenges in children with ASD has gained significant clinical and research attention (14, 15).

At present, there is still a lack of effective evidence in China regarding PROMPT® intervention for children with language and speech delay and ASD. Therefore, the central research objective of this study is to determine whether a motor-speech planning intervention via PROMPT® is more relatively effective than standard parent-mediated cognitive guidance in advancing both the structural linguistic stages (measured by S-S stages) and functional communicative spontaneity (measured by image description scores) in children with ASD.

## Materials and methods

### Patients

This retrospective analysis study was conducted at Yongkang Women and Children's Health Hospital. The data for the research subjects were sourced from ASD children with language and speech delay who received treatment from January 2023 to January 2025. To enhance methodological rigor and comparability, 62 patients receiving PROMPT®-based language training were matched in a 1:1 ratio with 62 patients receiving structured parent-mediated home training guidance using propensity score matching (PSM).

*Inclusion criteria:*

- Diagnosed as meeting the diagnostic criteria for ASD in the 5th edition of the Diagnostic and Statistical Manual of Mental Disorders in the United States;
- According to the Sign Significant Relations (S-S) assessment of language and speech delay, there is a sign of language and speech delay (17);
- Age range: 3–10 years old;
- First time receiving systematic treatment and rehabilitation training related to ASD;
- The caregiver of the child signs informed consent and actively cooperates and insists on completing the treatment;
- Complete clinical data.

*Exclusion criteria:*

- Merge genetic metabolic diseases and other neurological and psychiatric organic disorders;
- Children with severe trauma such as traumatic brain injury and fractures;
- Children with organic lesions such as hearing impairment and blindness;
- Caregivers of children with intellectual disabilities;
- Unable to adhere to 3-month intervention and follow-up for any reason.

### Intervention procedure

This study was designed as a comparative effectiveness analysis between two active intervention modalities. Both groups received structured language-related interventions, and the outcomes reflect the relative clinical gains of PROMPT®-based training compared to home-based guidance rather than the absolute effect of PROMPT® alone. The control group received a structured parent-mediated intervention (home training guidance). In this approach, rehabilitation specialists provided parents with professional counseling and training, following which the parents implemented language cognitive training in the home environment three times per week, with each session lasting 1 h. The PROMPT® group received PROMPT®-based language training from experienced, certified speech-language therapists. For the PROMPT® group, intervention was conducted twice a week (30 min per session) for 3-months by certified speech-language therapists. The intervention followed a strict hierarchical path of seven stages: 1) Tone (establishing tone perception via rhythmic shoulder tapping); 2) Voice control (breathing-vocal synchronization via abdominal pressure); 3) Mandibular control (using tongue depressors of 1 mm–3 mm thickness to stabilize jaw opening); 4) Lip control (shaping lip tension for bilabial sounds); 5) Tongue control (using spoons and yogurt for spatial positioning); 6) Ordered movement (utilizing metronomes to coordinate multi-organ syllable transitions); and 7) Rhythm (intonation and stress marking). This hierarchical approach ensures that foundational physiological control supports higher-level speech sequencing and prosody (details in Supplementary Table S1).

### Primary outcome (structural stages)

To provide a holistic evaluation of the intervention, a multi-dimensional assessment framework linking linguistic structure to communicative function was employed. Assessment of language comprehension and expression ability via the S-S method. This measures the structural developmental stages of the child's linguistic system, ranging from gestural symbols to complex grammatical rules. The S-S method was developed by the China Rehabilitation Research Center. The ability can be divided into five stages from weak to strong: Stage 1 is difficulty in understanding things and their states; Stage 2 is the formation of basic concepts of things (divided into three sub stages: Stage 2-1 is functional operation of things; Stage 2-2 is matching, and Stage 2-3 is selection); Stage 3 is the symbol stage of things (divided into two sub stages: Stage 3-1 is gesture symbol stage and Stage 3-2 is language symbol stage); Stage 4 is the stage of words and language rules (irreversible) (divided into two sub stages: Stage 4-1 is two word stage and Stage 4-2 is three word stage); Stage 5 is the grammar rules (divided into two sub stages: Stage 5-1 is active voice and Stage 5-2 is passive voice). Each stage will have an age corresponding to the normal level of children's abilities, and language rehabilitation therapists often use this assessment to check children's language abilities.

### Secondary outcome (functional spontaneity)

#### Assessment of image expression ability

This measures how the achieved structural gains translate into functional spontaneity when the child is required to describe real-world visual stimuli. During evaluation, each child was seated on a fixed stool and initially asked to describe the picture spontaneously. The presence of active expression was recorded; in instances where no spontaneous expression occurred, the evaluator described the image as “Dad holding the child” and prompted the child to repeat the phrase. The child's performance within a 3-min period was then rated according to established scoring criteria. The specific scoring criteria are as follows: 1 point: In the case of paraphrasing, the child cannot pronounce or imitate the mouth shape; 2 points: In the case of retelling, children can imitate the mouth shape, but there is no obvious pronunciation; 3 points: In the case of repetition, children can repeat single sounds such as “dad”, “hug”, etc.; 4 points: In the case of retelling, children can retell disyllabic words such as “dad”, “child”, etc.; 5 points: In the case of retelling, children can retell 3 syllables, such as “dad holding”, “holding the child”, etc.; 6 points: In the case of retelling, the child can fully retell “Dad holding the child”; 7 points: able to spontaneously describe the content on the picture with a single syllable; 8 points: able to spontaneously describe the content on the picture using disyllabic words; 9 points: Able to spontaneously describe the content on the picture using three syllables; 10 points: Able to spontaneously and fully describe the content of the image. Notably, in spontaneous descriptions, children only need to mention syllables related to the content of the picture to be counted as points, and do not necessarily need to say syllables from “dad holding the child”; Anything related to the content of the picture, such as “uncle”, “baby”, “uncle holding baby”, etc., can be counted as points; In the case of paraphrasing, it is necessary to mention the syllables in “Dad holding the child” in order to calculate the score.

### Statistical methods

Statistical analyses were performed using SPSS software (version 27.0; IBM Corp, Armonk, NY, USA). Categorical variables, including gender, primary guardian, education level, and S-S stages, are expressed as frequencies and percentages (n,%) and were compared using the chi-square test. Data distribution was assessed for normality through visual inspection (histograms and probability plots) and analytical tests (Kolmogorov–Smirnov and Shapiro–Wilk tests). Non-normally distributed continuous variables, such as age, are presented as medians and interquartile ranges (IQRs) and were analyzed using the Mann–Whitney *U* test. To mitigate potential confounding and selection bias, a 1:1 propensity score matching (PSM) was implemented using a logistic regression model. Covariates incorporated into the model included gender, age, primary guardian, guardian's educational level, and baseline Sign Significant Relations (S-S) developmental stages. Matching was executed using the nearest-neighbor method with a caliper of 0.05. The balance of covariates post-matching was evaluated using standardized mean differences (SMDs), with an SMD<0.15 considered indicative of acceptable inter-group balance. To address the baseline imbalance in age observed in the matched cohort, an age-stratified analysis was specifically conducted. For all analyses, a two-tailed P<0.05 was considered statistically significant.

## Results

**Figure 1:**
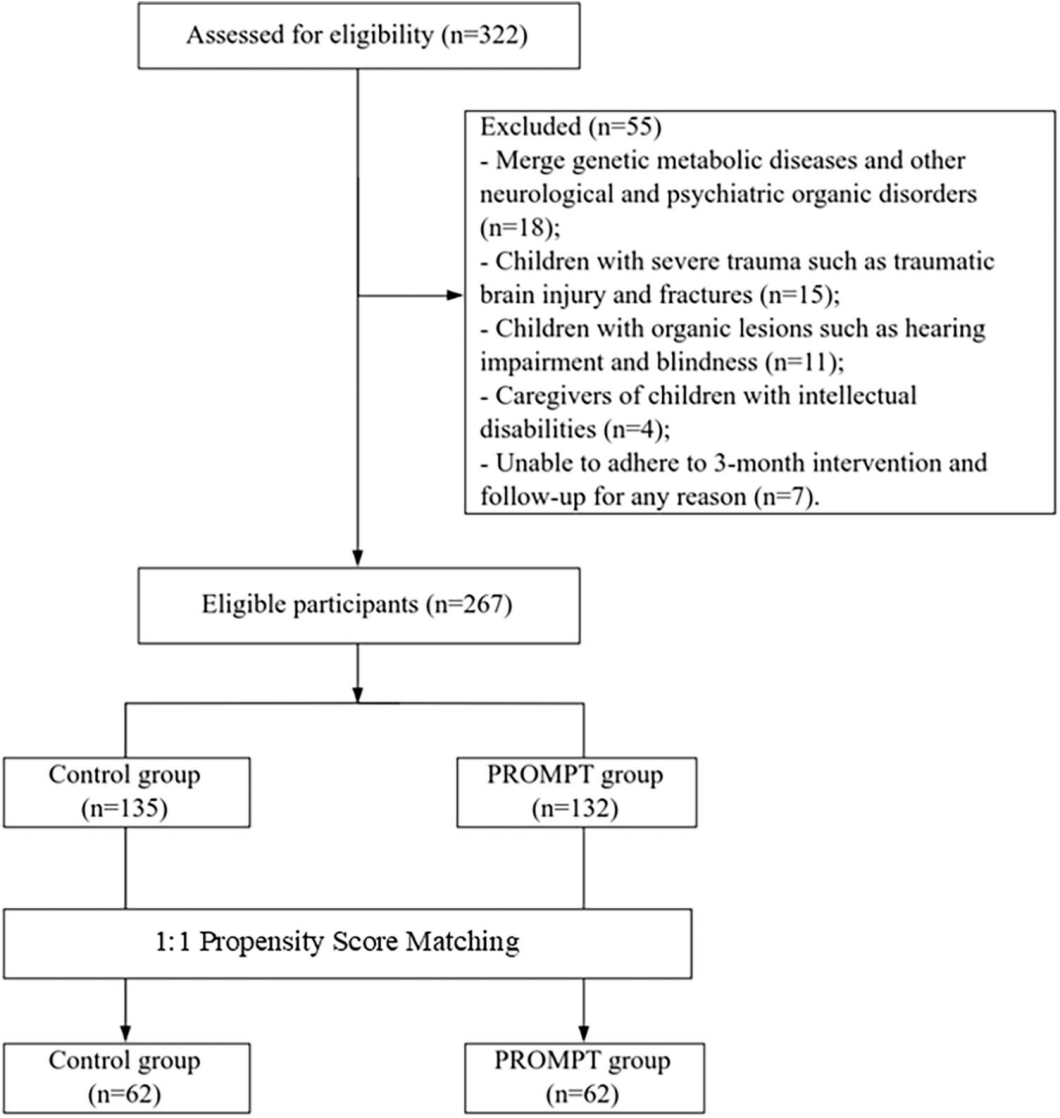
Flowchart of participant screening and 1:1 propensity score matching process.

A total of 322 children with ASD were initially screened. After excluding 55 children based on inclusion/exclusion criteria, 267 eligible participants remained. Following the 1:1 propensity score matching procedure, a final analytical cohort of 124 children (62 per group) was established (Figure 1). Post-matching analysis confirmed that the standardized mean differences for most baseline covariates were significantly reduced. While most variables achieved acceptable balance (SMD<0.15), age retained a slight imbalance. To ensure the robustness of the findings, this was addressed through subsequent age-stratified analysis (Table 1 and Supplementary Figure S1). No significant difference was observed in baseline demographic data between the two groups (P>0.05) (Table 1).

**Table 1:** Baseline demographic and clinical characteristics of the matched cohort.

Baseline data	PROMPT® group (*n* = 62)	Control group (*n* = 62)	*χ^2^/t*	SMD	*P*
Gender, *n* (%)			0.291	0.097	0.590
Male	31 (50.0)	34 (54.8)			
Female	31 (50.0)	28 (45.2)			
Age (years), Mean (SD)	5.48 ± 1.42	5.11 ± 1.29	1.519	0.273	0.131
Main guardian, *n* (%)			1.173	0.125	0.556
Mother	33 (53.2)	31 (50.0)			
Father	19 (30.6)	24 (38.7)			
Grandparents	10 (16.2)	7 (11.3)			
Educational level of primary guardians, *n* (%)			0.684	0.082	0.710
Primary school	6 (9.7)	9 (14.5)			
Junior high school	22 (35.5)	21 (33.9)			
High school and above	34 (54.8)	32 (51.6)			

Before intervention, there was no significant difference between the two groups in the distribution of S- S developmental stages for either language comprehension or expression (both *P* > 0.05). After the 3-month intervention, the distribution of S-S developmental stages differed significantly between the two groups, with the PROMPT® group showing a higher proportion of children in more advanced stages for both language comprehension$\left(\chi^{2}=12.795,P=0.025\right)$ and expression $\left(\chi^{2}=13.231,P=0.021\right)$ (Table 2).

**Table 2:** Distribution of S-S developmental stages between the two groups before and after intervention.

Time	Variables	PROMPT® group (*n* = 62)	Control group (*n* = 62)	*χ^2^*	*P*
Before intervention	Comprehension			2.186	0.702
	Stage 2-3	1 (1.6)	2 (3.2)		
	Stage 3-1	6 (9.7)	10 (16.1)		
	Stage 3-2	35 (56.4)	33 (53.2)		
	Stage 4-1	13 (21.0)	9 (14.5)		
	Stage 4-2	7 (11.3)	8 (12.9)		
	Stage 5-1	0 (0)	0 (0)		
	Expression			1.399	0.844
	Stage 2-3	1 (1.6)	2 (3.2)		
	Stage 3-1	9 (14.5)	10 (16.1)		
	Stage 3-2	38 (61.3)	39 (62.9)		
	Stage 4-1	8 (12.9)	8 (12.9)		
	Stage 4-2	6 (9.7)	3 (4.8)		
	Stage 5-1	0 (0)	0 (0)		
After intervention	Comprehension			12.795	0.025
	Stage 2-3	1 (1.6)	1 (1.6)		
	Stage 3-1	4 (6.5)	7 (11.3)		
	Stage 3-2	12 (19.4)	28 (45.2)		
	Stage 4-1	24 (38.7)	15 (24.2)		
	Stage 4-2	15 (24.1)	9 (14.5)		
	Stage 5-1	6 (9.7)	2 (3.2)		
	Expression			13.231	0.021
	Stage 2-3	1 (1.6)	2 (3.2)		
	Stage 3-1	8 (12.9)	10 (16.1)		
	Stage 3-2	17 (27.4)	30 (48.4)		
	Stage 4-1	16 (25.8)	15 (24.2)		
	Stage 4-2	17 (27.4)	4 (6.5)		
	Stage 5-1	3 (4.9)	1 (1.6)		

Before intervention, image expression ability showed no significant difference between the two groups (P>0.05) (Table 3). After intervention, image expression ability improved in both groups compared to baseline (P<0.05). The degree of improvement in the PROMPT® group was significantly higher than that in the control group $\left(\chi^{2}=15.984,P=0.043\right)$ (Table 3).

**Table 3:** Distribution of image expression scores between the two groups before and after intervention.

Scores	PROMPT® group (*n* = 62)	Control group (*n* = 62)	*χ^2^*	P
Before intervention			5.109	0.746
1 score	2 (3.2)	1 (1.6)		
2 scores	7 (11.3)	8 (12.9)		
3 scores	1 (1.6)	5 (8.1)		
4 scores	18 (29.0)	16 (25.8)		
5 scores	21 (33.9)	20 (32.3)		
6 scores	6 (9.7)	5 (8.1)		
7 scores	2 (3.2)	4 (6.5)		
8 scores	4 (6.5)	3 (4.8)		
9 scores	1 (1.6)	0 (0)		
10 scores	0 (0)	0 (0)		
After intervention			15.984	0.043
1 score	0 (0)	0 (0)		
2 scores	4 (6.5)	1 (1.6)		
3 scores	1 (1.6)	6 (9.7)		
4 scores	5 (8.1)	6 (9.7)		
5 scores	8 (12.9)	14 (22.6)		
6 scores	9 (14.5)	17 (27.4)		
7 scores	18 (29.0)	7 (11.3)		
8 scores	9 (14.5)	7 (11.3)		
9 scores	6 (9.7)	3 (4.8)		
10 scores	2 (3.2)	1 (1.6)		

**Figure 2:**
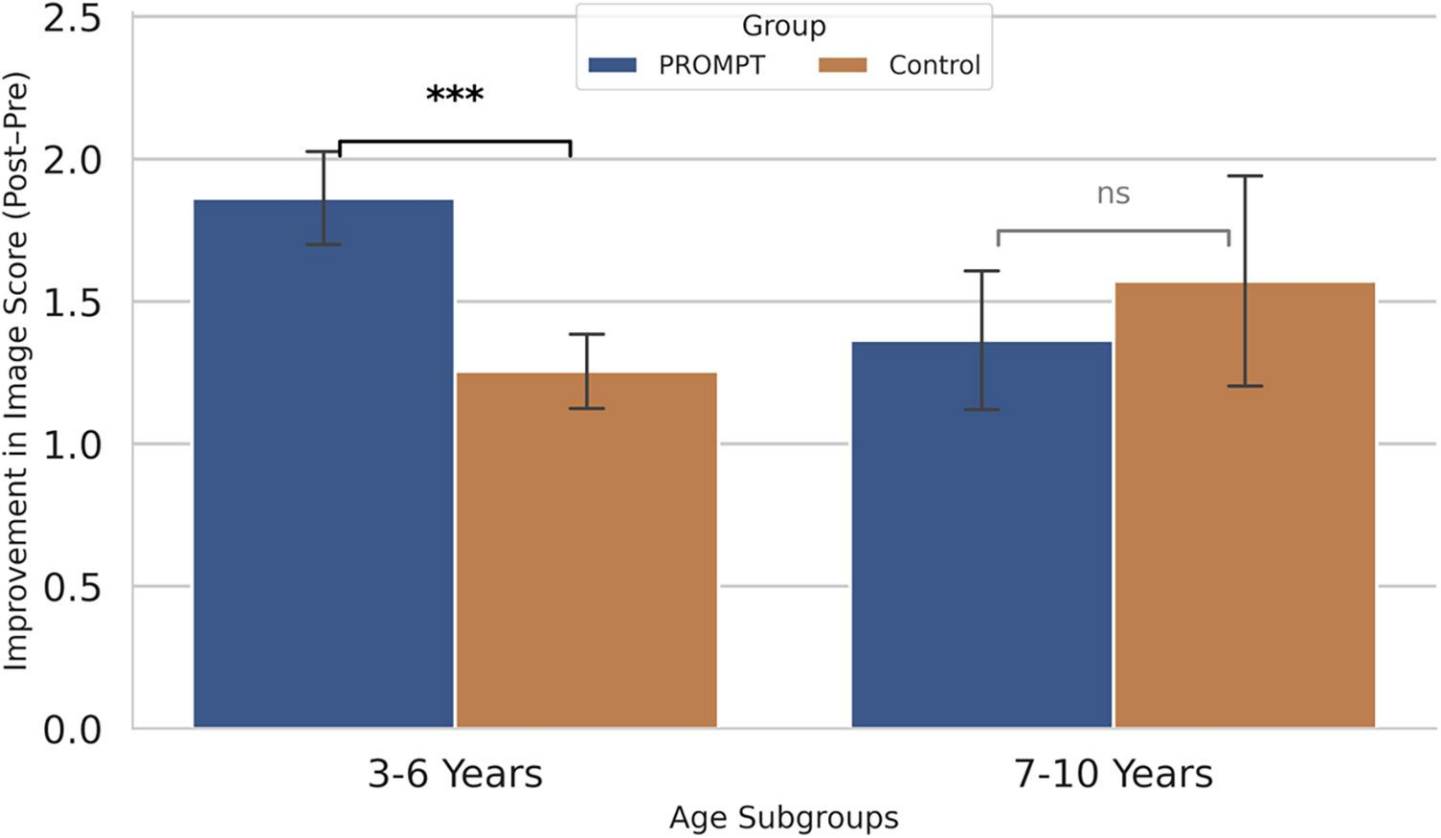
Heterogeneity of treatment effects on image expression score gains across age subgroups.

The subgroup analysis revealed distinct treatment responses across developmental stages (Table 4 and Figure 2). In the younger subgroup (3–6 years), the PROMPT® group achieved significantly greater gains compared to the control group in all core measures: S-S comprehension (Z=3.715,P<0.001), S-S expression (Z=3.048,P=0.002), and image expression scores (Z=2.482,P=0.013). Conversely, no statistically significant differences in treatment gains were observed between the two groups in the school-age subgroup (7–10 years) for any dimension (allP>0.05).

**Table 4:** Comparison of treatment outcome gains between the PROMPT® and control groups stratified by age subgroups.

Subgroup	Outcome	PROMPT® (Mean ± SD)	Control (Mean ± SD)	*Z*	*P*
	S-S comprehension	0.84 ± 0.83	0.27 ± 0.56	3.175	<0.001
3–6 Years	S-S expression	0.63 ± 0.80	0.13 ± 0.34	3.048	0.002
	Image score	1.86 ± 1.17	1.25 ± 0.97	2.482	0.013
	S-S comprehension	0.36 ± 0.50	0.57 ± 0.53	0.725	0.431
7–10 Years	S-S expression	0.36 ± 0.50	0.71 ± 1.11	0.371	0.767
	Image score	1.36 ± 0.81	1.57 ± 0.98	0.362	0.660

## Discussion

To our knowledge, this is a rare study in China on the effect of PROMPT® based language training on language and speech delay in children with ASD. The findings demonstrate that PROMPT®-based language training provides superior relative effectiveness compared to home training within a 3-month period. In contrast to traditional “top-down” behavioral interventions that primarily target social-communication symptoms, PROMPT® utilizes a “bottom-up” approach that prioritizes the underlying speech motor control process. Notably, the PROMPT® group received less total intervention time (1 h/week) than the control group (3 h/week). Despite this, they achieved significantly greater gains in both S-S stages and image expression scores This higher intervention efficiency is highly consistent with the results of international randomized controlled trials by Namasivayam et al. (15) and structural neuroplasticity studies by Fiori et al. (14). These data further validate that for children with ASD, direct physical manipulation of the articulators provides the essential sensory-motor feedback necessary to bypass the executive and motor planning dysfunctions frequently observed in this population.

CLINICALLY, ASD children with language and speech delay have been observed to have abnormal structural or functional development of their brain's speech motor network; For example, the entire chain of “plan execute feedback” cannot function properly in terms of regulation (18, 19). Thereby affecting the accuracy and stability of speech output, speech and rhythm output; In addition, these children also have abnormalities in touch, movement, and other aspects of their articulatory organs (lips, tongue, teeth, jaw, and soft palate); For some phonemes (such as d/t, m/n), it is difficult to pronounce and sometimes there may be confusion and missing sounds. Saying “ma” will be pronounced as “mai”, and saying “chi” will be pronounced as “ci” (20, 21). Our results showed that after the 3-month intervention, the PROMPT® group exhibited a more advanced distribution of S-S language comprehension and expression stages than the control group, with a higher proportion of children reaching higher developmental stages. Meanwhile, previous studies have shown that oral motor training can improve the language comprehension and expression abilities of children with ASD (22, 23). PROMPT®-based language training connects speech acts from three different dimensions: sensory perception, cognitive language, and socio emotional, to better promote the development of oral perception; This approach facilitated the control of uncoordinated movements in the jaw, lips, and tongue, while establishing new motor patterns to enable these children to acquire improved pronunciation and language expression abilities.

From a practical perspective, the effectiveness of PROMPT® in this study is anchored in its systematic 7-step hierarchical intervention path (12, 13). This framework provides clinicians with a clear, replicable logic: beginning with foundational physiological regulation (such as tone perception and breathing-vocal synchronization) and progressing to the stabilization of specific articulators like the mandible, lips, and tongue (12, 13). By addressing common oral-motor deficits in ASD—such as poor jaw stability or uncoordinated tongue-tip movement—through graded, tactile-kinesthetic cues, PROMPT® transforms abstract phonological targets into perceptible physical movements (14). This structural approach is particularly applicable to ASD children who struggle with traditional auditory-visual imitation, as it reduces the cognitive mapping effort required to initiate speech motor plans (13, 14).

The improvement in language comprehension observed in the PROMPT® group may be attributed to enhanced sensory-motor integration (13, 16). By providing synchronized tactile-kinesthetic, auditory, and visual feedback, PROMPT® facilitates the establishment of more stable neural phonological representations in children with ASD (16). According to the Motor Theory of Speech Perception, the ability to produce speech sounds is closely coupled with the ability to perceive and decode them (24); thus, mastering articulatory patterns through physical prompts can directly enhance the efficiency of linguistic processing (24). Furthermore, by improving articulatory coordination and making speech production more automated, PROMPT® reduces the “motor execution load” during communication (13). This allows the child to reallocate limited cognitive resources, such as attention and working memory, toward higher-level semantic processing and linguistic decoding, ultimately leading to improved comprehension stages (25).

Furthermore, our age-stratified analysis underscores a “critical window of opportunity” for PROMPT® intervention. The pronounced benefits observed exclusively in the 3–6 years subgroup suggest that tactile-kinesthetic prompts are most effective during early childhood, when speech motor planning and articulatory patterns are highly malleable. As children reach school age (7–10 years), speech motor habits become more established, potentially leading to the comparable relative effectiveness observed between PROMPT® and home-based guidance. These findings reinforce the clinical necessity of prioritizing PROMPT®-based language training for younger children with ASD to maximize developmental gains.

The S-S assessment was primarily selected as the primary outcome because it is a widely recognized standard clinical tool for evaluating language delay in China. Given the retrospective nature of this study, S-S results also represented the most consistently and comprehensively recorded data in our clinical archives. To address potential concerns regarding the categorical sensitivity of S-S stages over a 3-month period, we specifically incorporated a 10-level image expression score. This more granular scale allowed for the detection of fine functional improvements—ranging from sound imitation to spontaneous description—that might be overlooked by broader developmental stages, thereby providing a more precise evaluation of short-term intervention efficacy. The image expression ability score used in this study is based on the image expression ability score level of Chen et al. (26), which is further divided into 10 levels to provide a more detailed evaluation of children's functional expression ability. The number of spontaneously expressed cases in the PROMPT® group increased from 7 cases before treatment to 35 cases, which was higher than the control group (from 7 cases before treatment to 18 cases). Therefore, it is believed that PROMPT® language training is beneficial for children's expression ability through the use of auditory feedback (pronunciation) and tactile cues (manual, lip and tongue movements). Specifically, it is manifested in enabling children to more accurately understand content through pictures, improving their ability to control facial expressions, lip and tongue areas, and jaw areas, as well as enhancing their oral output through speech and motor planning and execution (27, 28).

Several limitations in this study should be acknowledged. First, the single-center, retrospective design and relatively small sample size may limit the generalizability of the findings to broader populations. Second, as a non-randomized study, potential selection bias cannot be entirely excluded, although propensity score matching (PSM) was utilized to enhance group comparability. Third, the image expression scale, while clinically useful for detailed functional evaluation, currently lacks established standardized reliability and validity. Regarding the intervention duration, the 3-month (12-week) period used in this study is widely recognized in speech-language pathology as a standard clinical cycle for evaluating functional changes in articulatory precision and motor planning. Given the current scarcity of evidence-based research on PROMPT® for ASD in China, these results provide critical preliminary evidence for its short-term relative effectiveness. However, it is acknowledged that this duration is primarily sufficient for observing early gains and may be insufficient to fully assess the long-term stability of improvements or complex neurodevelopmental trajectories. To address these constraints, future research should focus on the following aspects: 1) Conducting multi-center randomized controlled trials (RCTs) with larger cohorts to validate these findings and improve the reliability of the conclusions; 2) Extending the follow-up period to 6–12 months to assess the long-term persistence of therapeutic effects; 3) Employing more sensitive, specialized standardized language tests alongside neuroimaging techniques (such as fMRI) or electrophysiological methods to further elucidate the neural mechanisms of the PROMPT® intervention; 4) Performing refined stratified analyses based on ASD severity, developmental biomarkers, and long-term neurodevelopmental trajectories to clarify the optimal intervention window for different populations.

## Conclusion

In conclusion, PROMPT®-based intervention offers superior relative effectiveness compared to standard parent-mediated home training for speech-language delay in children with ASD. By prioritizing the reconstruction of motor-speech planning, this “bottom-up” approach not only resolves physical articulatory barriers but also facilitates significant cross-dimensional gains in language comprehension and functional spontaneity. Despite its short-term nature, this study fills a vital evidentiary gap in the Chinese clinical context and establishes a physiological-based framework for speech rehabilitation in ASD, providing a robust foundation for future longitudinal and neurological explorations.

## Data Availability

The original contributions presented in the study are included in the article/Supplementary Material, further inquiries can be directed to the corresponding author.

## Supplementary material

SUPPLEMENTARY FIGURE S1

Love plot illustrating the balance of baseline covariates before and after propensity score matching.
