# Correction: Comparative effectiveness of PROMPT-based language training vs. structured home-based training for language and speech delay in children with autism spectrum disorder

**DOI:** 10.3389/fped.2026.1843094

**Published:** 2026-04-09

**Authors:** Wei Hu, Qin Liu, Xuemei Fu, Yanmei Chen

**Affiliations:** Department of Children’s Rehabilitation, Yongkang Women and Children’s Health Hospital, Yongkang, Zhejiang, China

**Keywords:** autism spectrum disorder, children, language and speech delay, language training, PROMPT

Abstract

In the abstract, the text under the “Results” and “Conclusions” headings was erroneously interchanged. This has been corrected to read:

Results: After the 3-month intervention, the distribution of S-S developmental stages for language comprehension and expression differed significantly between the two groups, with the PROMPT group showing a higher proportion of children in more advanced stages than the control group (*P* < 0.05). In addition, image expression ability improved in both groups after intervention, and the improvement was greater in the PROMPT group than in the control group (*P* < 0.05).

Conclusions: PROMPT-based language training was associated with better outcomes than structured home-based training in children with ASD and language and speech delay, with greater improvement in language developmental stages and image expression ability.

The original version of this article has been updated.

