# Supplementary figures and images for: Comparative effectiveness of PROMPT®-based language training vs. structured home-based training for language and speech delay in children with autism spectrum disorder
Source: Front Pediatr. 2026 Mar 26;14:1726236. doi: 10.3389/fped.2026.1726236 (PMC13062323; doi:10.3389/fped.2026.1726236)

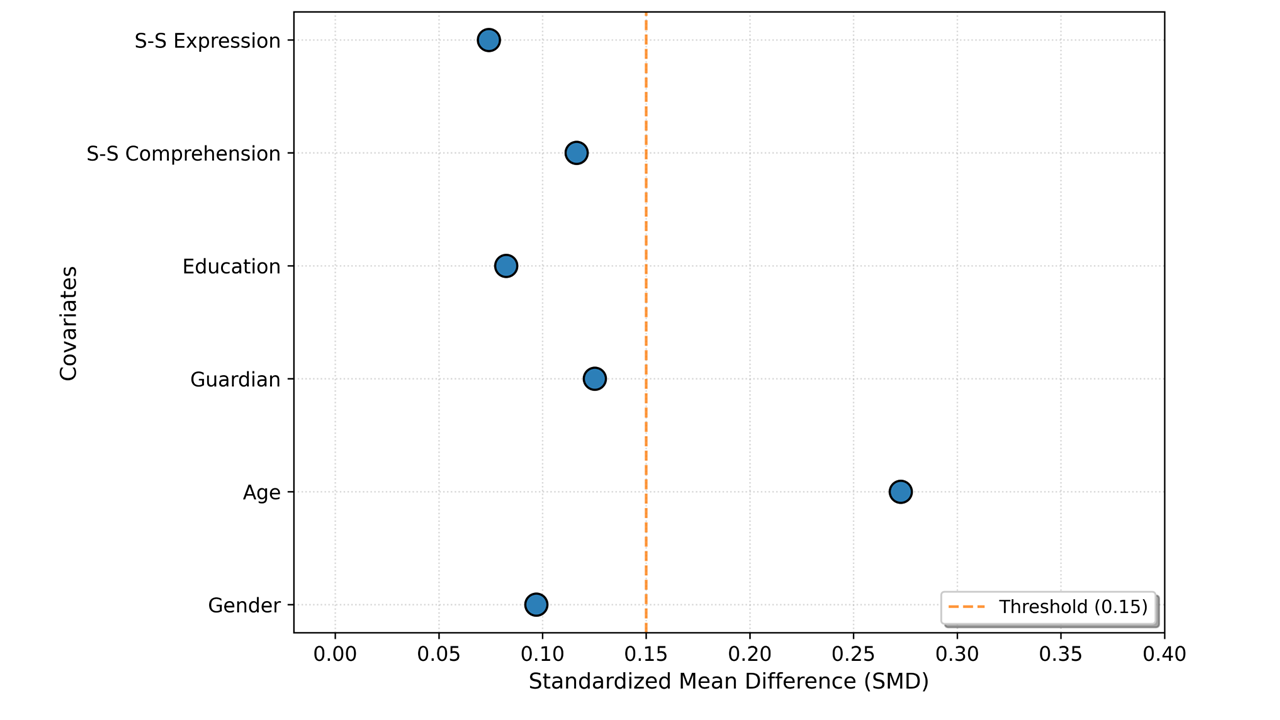

Supplement: Supplementary file 1 [file Image1.tif]
