# Supplementary material for: Comparative effectiveness of PROMPT®-based language training vs. structured home-based training for language and speech delay in children with autism spectrum disorder
Source: Front Pediatr. 2026 Mar 26;14:1726236. doi: 10.3389/fped.2026.1726236 (PMC13062323; doi:10.3389/fped.2026.1726236)
Supplement: Supplementary file 2 [file Table1.docx]

Supplementary Table 1. Two sets of intervention measures

| Stage | Target | Language Training Based on PROMPT | House-training |
| --- | --- | --- | --- |
| Step 1: Tone | Establish tone perception and preliminarily express simple emotional tones | 1. Precise tactile auditory linkage: transmitting tone and rhythm through rhythmic tapping of the shoulder (slow tapping when happy, fast tapping when surprised); Simultaneously touch the facial muscles (corners of the mouth, brow bones) with your fingers, allowing the child to "touch" the expression changes corresponding to the tone.   2. Individualized tone correction: To address the issue of flat tone, use the thumb to lightly support the chin and assist in raising and lowering the tone (gently lift when raising tone, lightly press when lowering tone), directly intervening in the movement of the vocal organs.  3. Instant feedback mechanism: Adjust the tone intensity in real-time through fingertip touch during pronunciation to avoid ineffective imitation. | 1. Daily tone interaction: When reciting nursery rhymes, one should exaggerate (such as using a warm voice for "Little Rabbit, Be Good~" and a fast voice for "Big Bad Wolf is Coming!").  2. Emotional scene connection: When happy, the tone is sweet, and when crying or pouting, the tone is low.  3. Language sense game, say "Hi, Hi, Hi" and try changing "Hi" to different tones. |
| Step 2: Voice control | Master the ability to regulate basic vocal intensity and duration | 1. Breathing Vocal Synchronization Training: Gently press the abdomen with your palm to feel the ups and downs of your breathing, accurately guiding the rhythm of "inhaling for 3 seconds holding your breath for 2 seconds vocalizing for 4 seconds", and controlling the duration of your vocalization through abdominal pressure feedback;  2. Precise regulation of vocal cord vibration: lightly touch the throat with fingertips to sense the strength of vibration (weak vibration when low, strong vibration when loud), and directly correct the problem of "too low/too loud sound";  3. Quick intervention for stuttering: Gently tap the lips and throat to quickly activate the vocal activation mechanism and reduce pauses that are difficult to express. | 1. Blow and suck game: Practice long exhalation with a light cloth strip or paper strip, and say "wow~" while exhaling.  2. Daily volume adjustment: Say "softly call mom" during meals and "loudly call dad" outdoors.  3. Animal vocal imitation: Learn from the kitten's "meow~" (soft and long sound) and the puppy's "woof!" (short and strong sound). |
| Step 3: Mandibular control | Stabilize the amplitude and strength of jaw opening and closing, support basic pronunciation | 1. Quantitative opening and closing training: Measure the amplitude of jaw opening and closing with fingers (target two finger width), and conduct a three-stage training of "passive assistance semi assistance autonomous control"; Ensure that the opening and closing amplitude is consistent each time.  2. Strength grading enhancement: Use tongue depressors of different thicknesses (1mm → 3mm) for children to bite and gradually increase the strength of jaw closure; Avoid "mixed sound" caused by jaw relaxation during pronunciation.  3. Pronunciation Movement Binding: Every time you perform an opening and closing movement, synchronize the sound of "ah (open) - wu (close)" to establish a direct association between "movement speech". | 1. Eating assistance training: When eating cookies, guide "open your mouth wide and bite - slowly close your mouth and chew", saying "open your mouth wide, ah~" and "close it, bite~".  2. Jaw game: Play "mouth robot", parents say the "on/off" command, children cooperate with actions and pronunciation.  3. Toy interaction: Use adhesive toys to actively bite and bite while counting "1, 2, 3". |
| Step 4: Lip control | Enhance lip movement flexibility and closure ability, accurately produce lip sounds | 1. Three dimensional shaping of lip shape: Gently pinch the lips with the thumb and index finger to assist in completing the three-dimensional movement of "spreading lips (i) - round lips (u) - closed lips (m)"; Each lip shape is held for 4-6 seconds, enhancing memory through fingertip touch.  2. Precise intervention for lip sound explosion: In response to the problem of "p/b" being pronounced as "m", gently press the lips with your fingers and quickly release them to assist in the explosion of airflow, directly correcting pronunciation errors.  3. Power pronunciation correlation: By providing lip tension feedback during whistling, the perception of "closing power=clarity of pronunciation" is strengthened. | 1. Lip game: Play "lip touching" (touching toys, fingers), say "touch it, close your mouth"; When blowing bubbles, say "round lips, blow bubbles~";.  2. Daily lip tone enhancement: Exaggerate pursed lips when saying "dad" or "mom".  3. Mouth shape imitation: Parents make "pout" and "grin" to guide imitation and send corresponding "u" and "i". |
| Step 5: Tongue Control | Enhance the accuracy and range of tongue movement, and improve the pronunciation of tongue tip/tongue surface sounds | 1. Tongue visualization positioning: Use a tongue depressor to mark key positions such as the tip of the tongue touching the gingiva (d/t) and the retraction of the tongue surface (g/k), and use your fingers to gently push the tongue surface to assist in reaching precise points.  2. Immediate correction of errors: For "n/l indistinguishable", gently lift the tip of the tongue with fingertips against the gingival hair "n", gently push the tongue side to move the hair "l", and compare the perceptual differences.  3. Full range exercise training: Design a combination of "forward extension, upward lifting, and lateral movement" movements, with 3 groups each time, to systematically expand the range of tongue movement. | 1. Tongue game: Dip a spoon in yogurt and guide the tongue to extend and lick (extend forward), the tip of the tongue to lick the corners of the mouth (move sideways), and say "tongue long, lick".  2. Training when brushing teeth: When brushing the tip of the tongue, say "tongue up, brush above", and when brushing the surface of the tongue, say "tongue flat, brush in the middle".  3. Food interaction: When eating noodles, roll the tip of your tongue and say "roll the tip of your tongue, roll the noodles". |
| Step 6: Ordered Movement | Coordinate lip, tongue, and jaw movements to complete continuous syllable/vocabulary pronunciation | 1. Multi organ collaborative training: Use the "hand guided lip+tongue+jaw" joint movement to train syllables such as "dad dad" (tongue tip lip transition) and "apple fruit" (tongue surface lip transition); Ensure smooth movement connection through tactile synchronization prompts.  2. Initials vowel bridging: Use your fingers to first fix the tongue position of the initial consonant (such as closing your lips when "b"); Further guide the transition to vowel mouth shape (with lips spread out when "α"); Strengthen syllable connections without stuttering.  3. Rhythm visualization: Use a metronome and touch your lips with your fingers, press "1 beat, 1 syllable" to control the pronunciation rhythm and avoid missing/rushing sounds. | 1. Children's song practice: Sing "Daddy's Daddy calls Grandpa", clap hands and rhythm to emphasize clear syllables.  2. Daily vocabulary repetition: When saying "baby eat" or "mom hug", slow down and guide the imitation of syllable transitions.  3. Item naming dragon: Play "fruit dragon", say "apple fruit → fragrance banana", break down the syllables and say. |
| Step 7: Rhythm | Mastering intonation, rhythm, stress, and pauses to improve language fluency | 1. Physiological regulation of intonation: Use fingers to gently lift the lower jaw to assist in raising the tone of interrogative sentences ("? ↑"), and gently press the lower jaw to assist in lowering the tone of declarative sentences (". ↓"), converting intonation changes into perceptible jaw movements.  2. Accurate stress marking: Gently tap the back of the child's hand with the palm (heavy tap when stressed, light tap when light), combined with throat vibration perception, to reinforce the stress position.  3. Pause structured training: Touch the desktop with your fingers to mark pause points ("bird fly, fly high"), synchronously control your breathing rhythm, and avoid language flow disorder. | 1. Sing/recite nursery rhymes: Sing "Two Tigers" and clap to emphasize the stress of "running fast" and "really strange".  2. Story reading: When reading picture books, imitate character dialogues with different tones (gentle little bear, rough big bad wolf).  3. Rhythm game: Play "Clap your hands and say sentences", say "Today - the weather - it's really nice", and press the corresponding word to clap. |
